# Evaluation of native macroalgae species of the Southeast U.S. and Caribbean for use in integrated multi-trophic aquaculture (IMTA)

**DOI:** 10.1007/s10499-026-02441-1

**Published:** 2026-02-10

**Authors:** Haley L. Lasco, Hilary G. Close, Ronald H. Hoenig, Phillip R. Gillette, Daniel D. Benetti, John D. Stieglitz

**Affiliations:** 1 Department of Marine Biology and Ecology, Rosenstiel School of Marine, Atmospheric, and Earth Science, University of Miami, 4600 Rickenbacker Causeway, Miami, FL 33149 USA; 2 Department of Ocean Sciences, Rosenstiel School of Marine, Atmospheric, and Earth Science, University of Miami, 4600 Rickenbacker Causeway, Miami, FL 33149 USA; 3 Present Address: South Carolina Department of Natural Resources (DNR), 217 Fort Johnson Rd., Charleston, SC 29412 USA

**Keywords:** Macroalgae, Seaweed, IMTA, Marine finfish, Aquaculture, Sustainability

## Abstract

This study assessed the comparative performance of candidate marine macroalgae species under integrated multi-trophic aquaculture (IMTA) culture conditions in a system culturing yellowtail snapper (*Ocyurus chrysurus*) at a commercial-scale biomass density. The nutrient-rich effluent water from the marine finfish culture tank supplied replicated macroalgae raceways containing four candidate species of macroalgae (*Agardhiella subulata*, *Caulerpa racemosa*, *Gracilaria caudata*, and *Ulva lactuca*) native to the Southeast U.S. and Caribbean regions. Water temperature, dissolved oxygen (DO), salinity, alkalinity, pH, phosphate, and total ammonia nitrogen (TAN) readings were sampled daily, and dissolved CO_2_ was calculated over the course of the trials. Each species of macroalgae was analyzed for protein, fat, fiber, ash, minerals, and metals. Additionally, the carbon and nitrogen content and stable isotope ratios of the nutritionally enriched (i.e., “fortified”) macroalgae (“seaweed”) species were analyzed. Results provide novel insights on macroalgae biomass levels under specific hydraulic retention times (HRT) that allow for the reduction of TAN levels in fish effluent water below detectable levels. Specifically, *Agardhiella subulata* at a density of 6.73 kg m^−3^, lowered TAN levels in fish effluent water below detectable limits under a hydraulic residence time (HRT) of 54 min from average starting concentrations of 0.04 mg/L TAN. Compositional analyses indicated *Caulerpa racemosa* had the highest protein content (25.49%) and *Ulva lactuca* had the highest carbohydrate content (61.48%) of the macroalgae species tested. Calculated dissolved CO₂ concentrations of effluent water before and after the macroalgae culture tanks and the carbon-to-nitrogen ratio of macroalgae samples indicated that *Ulva lactuca* up took and incorporated carbon most efficiently. The results of this project provide novel information that will help improve economic and environmental sustainability for existing and prospective marine finfish aquaculture operations throughout the focal regions and allow producers to make well-informed decisions on candidate species of macroalgae for marine IMTA applications.

**Supplementary Information:**

The online version contains supplementary material available at 10.1007/s10499-026-02441-1.

## Introduction

Marine finfish aquaculture in the United States of America (USA) has great potential to aid in increasing domestic seafood supply and reducing the country’s reliance on imported seafood. Two of the major issues with marine aquaculture in the USA are the regulatory/permitting processes and the social license challenges associated with the industry (Rust et al. 2014). One promising path to better social acceptance of marine aquaculture and improved economic and environmental viability of operations is the concept of developing integrated farms that capitalize on value chains across multiple trophic levels. Such aquaculture operations are commonly referred to as integrated multi-trophic aquaculture (IMTA), which can be implemented in land-based and open ocean systems (Tanaka et al. 2020). IMTA is the concept of growing multiple organisms from different trophic levels in one system (Chopin et al. 2001; Lohroff et al. 2021; Neori et al. 2004; Paul and Nys 2008), whereby production from the initial feed energy input(s) is maximized. The IMTA methodology allows for significant reductions, and in many cases, elimination of nutrient loading in effluent water from fed aquaculture operations, thereby allowing the water to be safely discharged or recirculated while growing a secondary biomass for various market uses (Zhu et al. 2025). Macroalgae have been identified as an option for use in IMTA due to their ability to uptake nitrogen, carbon, phosphate, and sulfate (Jasmin et al. 2020). Macroalgae are an advantageous choice for bioremediation, as their culture is not limited to contained vessels as it typically is with microalgae. For instance, extensive macroalgae culture is conducted globally in open systems in coastal and offshore waters (de Gaillande et al. 2017; Kim et al. 2019b).

Some of the most prevalent nutrients produced from finfish aquaculture systems, which macroalgae can help reduce, are ammonium (NH_4_^+^), phosphate (PO₄^3^⁻), and sulfate (SO₄^2−^). Under optimized growth conditions, one study found that *Ulva lactuca* stocked at 0.8 kg m^−2^ was able to assimilate 88% of the nitrogen in the effluent water of a commercial finfish facility (Ben-Ari et al. 2014). It was also found that *Caulerpa racemosa* grew at maximum rates under relatively high phosphate concentrations (Harwanto et al. 2020). Another study, by Narvarte et al. (2025), found that *Caulerpa microphysa* had the highest phosphate uptake when compared to five other commercially relevant seaweed species. These studies suggest that there is potential for *Caulerpa racemosa* to be a suitable candidate for the bioextraction of both phosphate and nitrogen.

Much of the history of macroalgae farming in the USA is with temperate species of kelp and *Porphyra* in states such as Washington, Alaska, and Maine (Kim et al. 2019a). These farms are supported locally because they provide food and help with reducing eutrophication in coastal waters (Kim et al. 2019b). While these states have strong familiarity with a variety of kelp species, there is little farming of tropical seaweed species in the USA. The USA has a unique position that allows southern coastal states such as Florida, Alabama, Louisiana, Texas, and Mississippi to grow local tropical seaweeds. The four species of macroalgae used in this study were identified in previous market research to be successful candidates for the seafood industry (Leandro et al. 2019), yet their suitability for incorporation in IMTA applications of high-value marine finfish species has not been fully evaluated.

End-use markets for seaweed are numerous and include human consumption, pharmaceuticals, market potential of extracts, and carbon sequestration and its pertinence to the blue economy (i.e., “carbon credits”) by way of restorative aquaculture. Macroalgae and microalgae have been looked at as additives and replacements in fish and terrestrial agriculture feed. *Ulva lactuca* has shown promise as a partial replacement for fish meal in fish feed. A study by Shpigel et al. found that there was no negative effect on seabream consuming the algae substitute diet; they were receiving all the necessary amino acids when 35% of the fish meal in the diet was replaced with *Ulva lactuca* (Shpigel et al. 2017). Additionally, the *Ulva lactuca* used in the Shpigel et al. (2017) study was from an IMTA system and contained 26–37% protein versus 8.5–18% protein in their wild counterparts (Shpigel et al. 2017; Padua et al. 2004; Yaich et al. 2011). Laramore et al. found that 25% of a commercial shrimp feed could be replaced with *Ulva lactuca* and maintain the same production success (Laramore et al. 2018). In general, when compared with other protein replacements in aquafeeds, seaweed shows great potential (Gu et al. 2016; Fleurence et al. 2012).

The main objectives of the present study were to quantify and characterize the bioextractive capacity and market potential of four macroalgae species in an integrated multi-trophic aquaculture (IMTA) system stocked with a commercially relevant marine finfish species at a relevant culture density (yellowtail snapper (*Ocyurus chrysurus*), stocked at 26 kg m^−3^). This study represents one of the first known studies comparing the side-by-side aquaculture performance of multiple macroalgae species in a pilot-scale IMTA system in the Southeast U.S. and Caribbean regions.

## Materials and methods

A pilot-scale Integrated Multi-Trophic Aquaculture (IMTA) system was developed at the University of Miami Experimental Hatchery (UMEH) in Virginia Key, FL, to evaluate the comparative performance of four candidate macroalgae species cultivated under consistent marine finfish effluent conditions. The primary objective was to assess species-specific differences in bioextractive capacity, nutritional value, and market potential when cultured under a standardized nutrient regime representative of commercial-scale marine finfish operations.

**Fig. 1:**
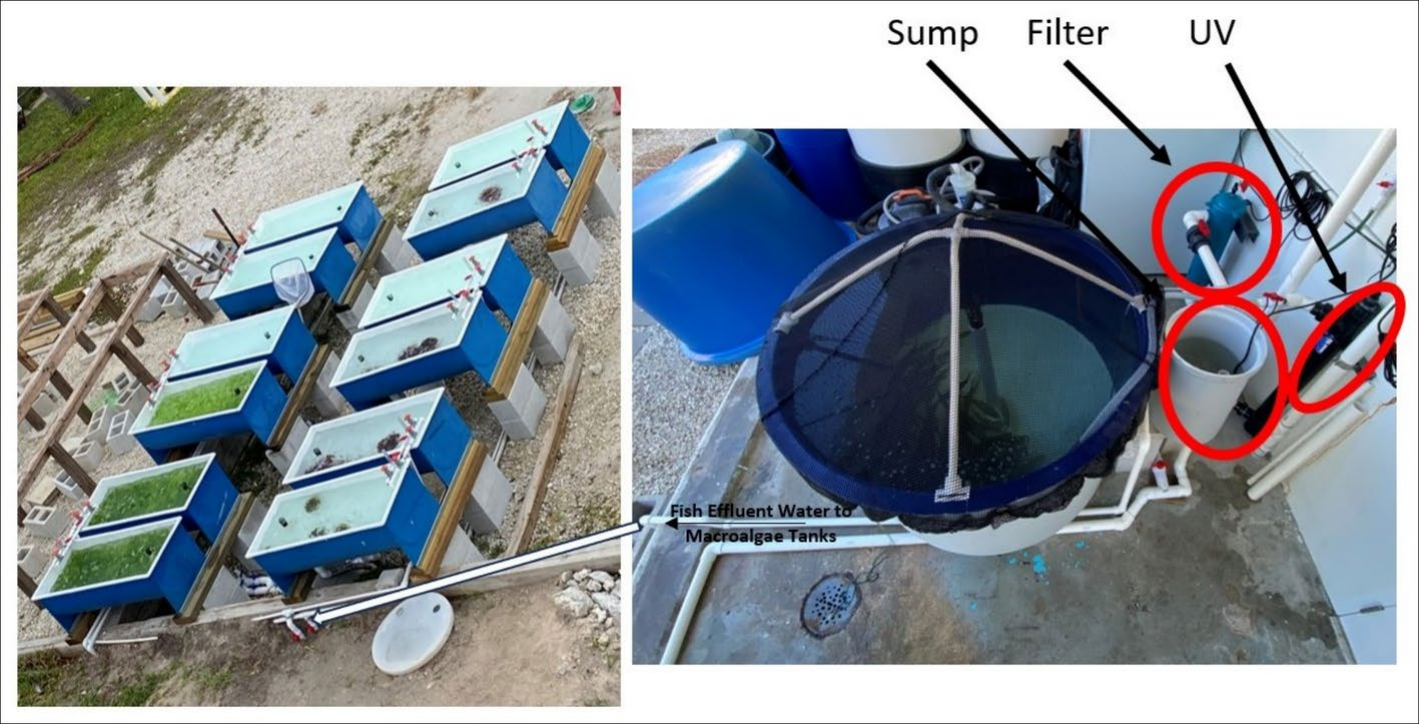
Photo/diagram of the pilot-scale IMTA system used in this study

The flow-through IMTA system was designed to provide a single, uniform source of nutrient-rich effluent generated by a 1 m^3^ culture tank (1.22 m diameter × 0.91 m depth) stocked with yellowtail snapper (*Ocyurus chrysurus*) at a commercial production density (26 kg m⁻^3^; mean weight = 152 ± 5 g; total length = 23.0 ± 0.2 cm). Fish were maintained using filtered, UV-sterilized seawater at ambient temperature, with supplemental oxygen provided as needed. Effluent from the fish tank passed through a particulate settling sump and was subsequently distributed evenly to 12 downstream macroalgae culture raceways via a common pump manifold (Fig. 1).

Each of the 12 macroalgae tanks (180 L capacity) received the same effluent water and represented a replicated experimental unit for assessing macroalgae performance. Four macroalgae species were tested, with three replicate tanks per species, enabling controlled comparisons across species under identical input conditions. Each tank had a flow control valve set at 0.95 gpm (3.33 Lpm), providing a hydraulic retention time of approximately 1 h. Aeration was supplied via bottom air lines to maintain macroalgal suspension. Effluent exited each tank through a top standpipe and was collected at the base of each tank for sampling. The macroalgae culture system operated outdoors under full sunlight and natural photoperiod. Combined effluent from all macroalgae tanks was collected in a centralized sump and discharged via an onsite disposal well. All aspects of the work involving live fish were conducted in accordance with University of Miami Institutional Animal Care and Use Committee (IACUC) Protocols #20–137 and #23–131.

There were two green macroalgae used, *Caulerpa racemosa* and *Ulva lactuca*, and there were two red macroalgae used, *Agardhiella subulata* and *Gracilaria caudata*. The *Agardhiella subulata*, *Gracilaria caudata*, and *Ulva lactuca* were obtained from the UM National Resource for *Aplysia*, while the *Caulerpa racemosa* was collected from local waters. Prior to initiating the experimental trials, all macroalgae were rinsed and epiphytes were removed. The algae were then placed into the system’s algae tanks, supplied from the fish tank, for acclimation which averaged 1–2 weeks in duration. The trial began once the macroalgae stabilized in color, structure, and growth.

Macroalgae stocking densities for each species were based on prior knowledge of potential growth rates and were selected to ensure that final harvest densities and experimental endpoints would not be compromised due to potential self-shading that can occur in macroalgae culture tanks when densities are too high. All weights represent wet weights of macroalgae. There was a total of 450 g of *Agardhiella subulata* to begin, so it was split into three tanks at 150 g per tank. The total *Caulerpa racemosa* starting biomass was 1338 g, split across three tanks (~ 300–500 g per tank). The *Gracilaria caudata* was stocked at 100 g per tank. The *Ulva lactuca* was stocked based on growth rates found during acclimation; the *Ulva lactuca* grew very fast, so to reduce the potential for self-shading and growth rate reductions before the end of the trial, the starting stocking density of *Ulva lactuca* was 10 g per tank. The experimental macroalgae culture duration (14-day culture cycle) was selected to allow enough time for the macroalgae to remove nutrients, but short enough to avoid self-shading issues that could occur at higher densities that could potentially confound experimental endpoints across the 180 L systems based on the macroalgae growth rates.

During the 2-week trial, a single, homogenized fish effluent source, distributed uniformly to all macroalgae tanks, was sampled daily at the common inlet point to each of the replicated macroalgae tanks to represent the baseline nutrient input. Seawater entering the fish culture tank had undetectable TAN concentrations and was not nutrient-rich to begin with. Concurrently, the effluent water from each of the individual macroalgae tanks was then sampled, with water sampling occurring each day at mid-day (~ 12:00 p.m.) to assess species-specific nutrient removal and performance. Mid-day was chosen so the algae had sufficient time to photosynthesize and for their respiration from the night to clear from the water. Water quality parameters were taken daily of dissolved oxygen (DO) (YSI ProODO, Pentair, Apopka, FL, USA), temperature (YSI ProODO, Pentair, Apopka, FL, USA), salinity, alkalinity (Hanna Alkalinity checker, Hanna Instruments, Smithfield, RI, USA), pH (Thermo Scientific Orion Star A221), total ammonia nitrogen (TAN) (Salicylate method 8155, Hach, Loveland, CO, USA), and phosphate (Phosphate reagent set Method 8178, Hach, Loveland, CO, USA). Dissolved CO₂ was calculated from alkalinity, pH, and temperature using a CO₂ calculator informed by Millero and Prieto (2002). Every 3 days, algae were collected with a mesh net, shaken dry (approximately five shakes of the net), and put into a plastic collection bucket to determine their wet weight. Once the weight was obtained, a sample of approximately 100 g from the most distal growth of the macroalgae was collected, rinsed with reverse osmosis (RO) water, and frozen for later analysis.

At the end of the 2-week trial, the total amount of each macroalgae was collected from each tank, cleaned of any obvious epiphytic growth, shaken dry, weighed, put in a bag, and stored in the freezer (–20 °C). Shortly after, they were thawed and put in a drying oven at 105 °C for 24–36 h. Once dried, as confirmed by obtaining weights of samples that did not differ after 2 consecutive days, the macroalgae were pulsed in a sterilized food processor until they were a fine powder. Prepared dry samples were analyzed for proximate analysis with carbohydrates and calories, fatty acid profile, amino acid profile, calcium, iron, magnesium, potassium, phosphate, lead, mercury, arsenic, and cadmium. The total carbohydrate was calculated from the moisture, protein, ash, and fat percentage data. All the macroalgae tissue compositional analyses were conducted by New Jersey Feed Labs, Inc. (Trenton, NJ, 08638, USA). The fats were determined using ether extraction of lipids, the protein was determined with nitrogen by combustion, the fiber was found by acid/alkali digestion, and ash was determined by the loss on ignition (New Jersey Feed Labs, Inc., Trenton, NJ, 08638, USA). For all the nutrient content obtained from analytical results, the percentages and ppm are in regard to the dry weight of the seaweed provided. The fats were provided on a relative basis where each fatty acid is expressed as a percentage (%) of the total amount (Table 2).

Macroalgae samples collected for elemental analysis were thoroughly rinsed with RO water, squeezed to rid the sample of any extra water, put into individual plastic bags, and lyophilized following initial freezing in a − 80 °C freezer. Once the samples were lyophilized, they were homogenized into fine powder using an isopropanol cleaned agate mortar and pestle. Exact weights of the powder were weighed into a tin capsule and analyzed. Analysis of carbon and nitrogen was conducted in the Laboratory for Marine Organic and Isotope Geochemistry at the University of Miami using a Thermo Flash Elemental Analyzer connected to a Thermo Conflo IV and MAT 253 isotope ratio mass spectrometer.

Data were analyzed using either an analysis of variance (ANOVA) or an analysis of covariance (ANCOVA), following testing of test-specific assumptions regarding distribution and homogeneity of variances. Differences between means were determined significant at *⍺* = 0.05. Water quality data from each macroalgae culture tank, including dissolved oxygen, alkalinity, phosphate, pH, TAN, and carbon dioxide, were compared across macroalgae species both in terms of incoming water (i.e., nutrient-enriched effluent) and outgoing water from each macroalgae tank. The ANCOVA provided the strength of the relationship between the parameter and the macroalgal species, as well as the parameter over the trial length. The data obtained from the compositional analysis were from a single homogenized batch sample of each macroalgae species, and therefore, comparative statistical analyses were not able to be utilized on these data. The resulting data from the nutritional analyses provide valuable information about each species of macroalgae and are highly relevant to this study and the industry alike. The ANCOVA analysis on the elemental data provided the relationship strength between the C:N ratio and the algal species and the C:N ratio over the trial length.

## Results

### Nutrient content

The total carbohydrates calculated were 42.35% for *Agardhiella subulata*, 48.7% for *Caulerpa racemosa*, 53.86% for *Gracilaria caudata*, and 61.48% for *Ulva lactuca*. *Agardhiella subulata* had the lowest carbohydrate content, and *Ulva lactuca* had the highest carbohydrate content. The macroalgae species with the highest protein content was *Caulerpa racemosa*, with 25.49%, and the species with the lowest amount of protein was *Ulva lactuca* with 13.56% (Table 1).

**Table 1:** Proximate analysis of all four macroalgae species; in percentage of total biomass or ppm of dry weight. *Agardhiella subulata*, *Caulerpa racemosa*, *Gracilaria caudata*, *Ulva lactuca*

	***A. subulata***	***C. racemosa***	***G. caudata***	***U. lactuca***
Moisture %	4.93	0.78	2.56	9.45
Protein (crude) %	22.66	25.49	21.22	13.56
Fat (AH) %	3.92	4.81	2.93	2.49
Total carbohydrate %	42.35	48.7	53.86	61.48
Fiber (crude) %	5.46	23.81	6.01	7.82
Ash %	26.14	20.22	19.43	13.02
Calcium %	0.55	0.56	0.25	0.43
Phosphorous %	0.14	0.2	0.15	0.13
Magnesium %	0.79	0.28	0.21	3.73
Iron (ppm)	33.6	66.2	26.8	30.2
Potassium %	6.53	0.69	7.77	1.03
Arsenic (ppm)	8	11.4	7.3	4.8
Lead (ppm)	< 0.05	0.7	< 0.05	< 0.05
Mercury (ppm)	< 0.05	< 0.05	< 0.05	< 0.05
Cadmium (ppm)	0.16	< 0.05	0.12	0.08

**Fig. 2:**
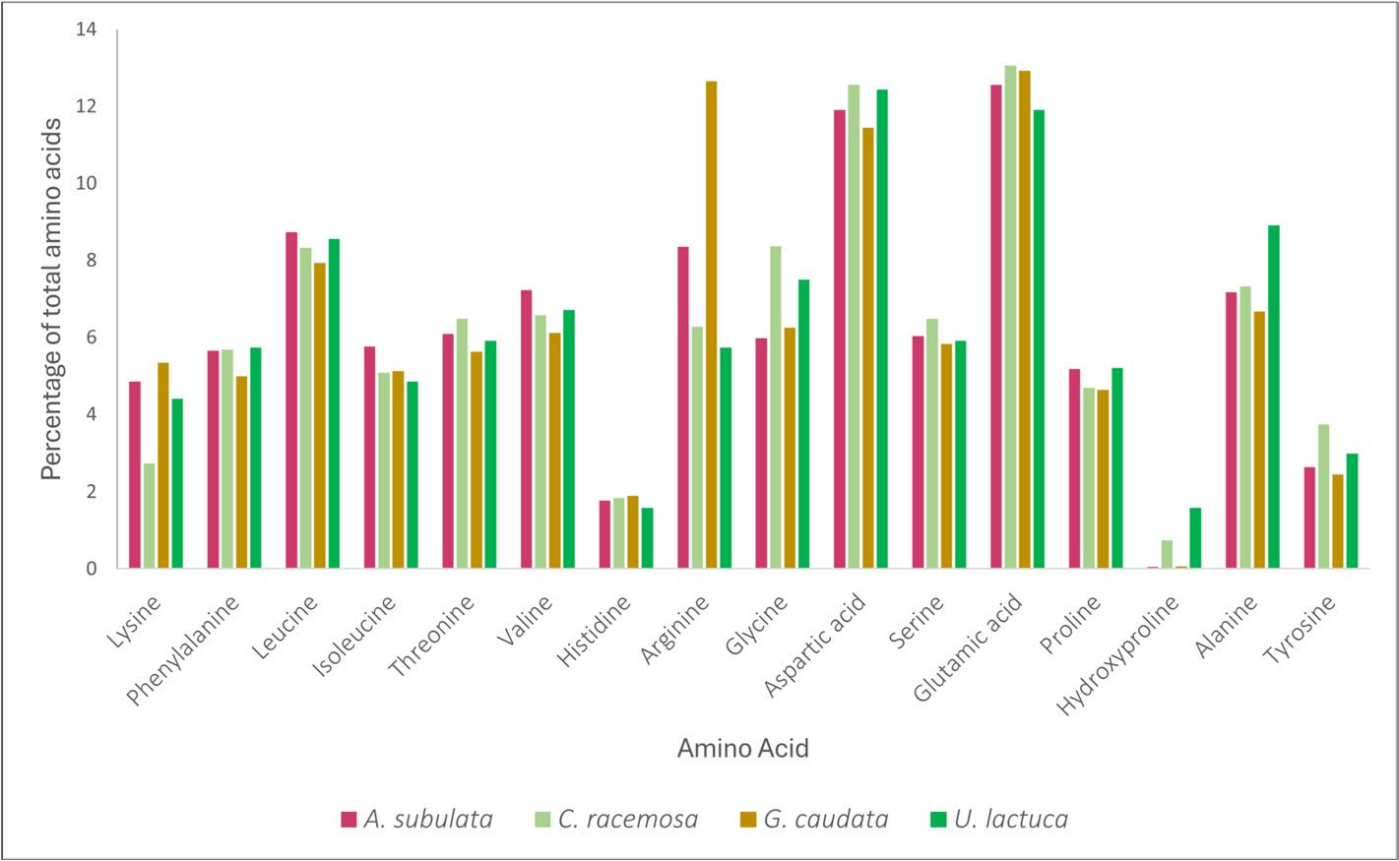
Amino acid type and the percentage of each within the total amino acids in each macroalgae species

The omega-3 and 6 data indicated that *Caulerpa racemosa* contained the most omega-3 with 19.52% of all fatty acids in the sample being omega-3, followed by *Agardhiella subulata* (12.67%), *Ulva lactuca* (10.10%), and finally *Gracilaria caudata* (2.79%) (Table 2). The omega-6 content was quite different from omega-3 with *Gracilaria caudata* leading with 33.39% of all fatty acids being omega-6, then *Agardhiella subulata* (17.21%), *Caulerpa racemosa* (11.55%), and finally *Ulva lactuca* (3.38%) (Table 2). Additionally, the amino acid analysis identified the specific amino acid concentrations in each macroalgae species (Fig. 2). The amino acid analysis indicated that histidine and hydroxyproline were in the lowest concentrations of all amino acids across all species of macroalgae, while leucine, aspartic acid, and glutamic acid were in the highest concentrations.

**Table 2:** Polyunsaturated fatty acids (PUFA), omega–3, and omega–6 content of each species, with relative % out of 100% fatty acids, and based on sample size. The sample size of *Agardhiella subulata* (As) was 90 g dry weight (DW), *Caulerpa racemosa* (Cr) was 21 g DW, *Gracilaria caudata* (Gc) was 46 g DW, and *Ulva lactuca* (Ul) was 137 g DW

	***A. subulata* (relative basis %)**	***A. subulata* (sample basis %)**	***C. racemosa* (relative basis %)**	***C. racemosa* (sample basis %)**	***G. caudata* (relative basis %)**	***G. caudata* (sample basis %)**	***U. lactuca* (relative basis %)**	***U. lactuca* (sample basis %)**
PUFA’s	12.17	0.26	4.6	0.12	2.37	0.03	0	0
Omega 3	12.67	0.27	19.52	0.49	2.79	0.04	10.10	0.11
Omega 6	17.21	0.36	11.55	0.29	33.39	0.46	3.38	0.04

### Minerals and metals

The compositional analysis provided data on minerals present in each macroalgae species. *Caulerpa racemosa* had the highest calcium percentage of 0.56%, *Ulva lactuca* had the highest magnesium percentage of 3.73%, *Gracilaria caudata* had the highest potassium percentage of 7.77%, and all species had between 0.1 and 0.2% of phosphorus (Table 1). Of the metals analyzed, *Caulerpa racemosa* contained the highest content of iron, arsenic, and lead with 66.2 ppm, 11.4 ppm, and 0.7 ppm, respectively, which are dry weight results (Table 1), while *Agardhiella subulata* had the highest cadmium level of 0.16 ppm, and all species were under 0.05 ppm in mercury content (Table 1).

### Water quality

#### Total ammonia nitrogen (TAN)

**Fig. 3:**
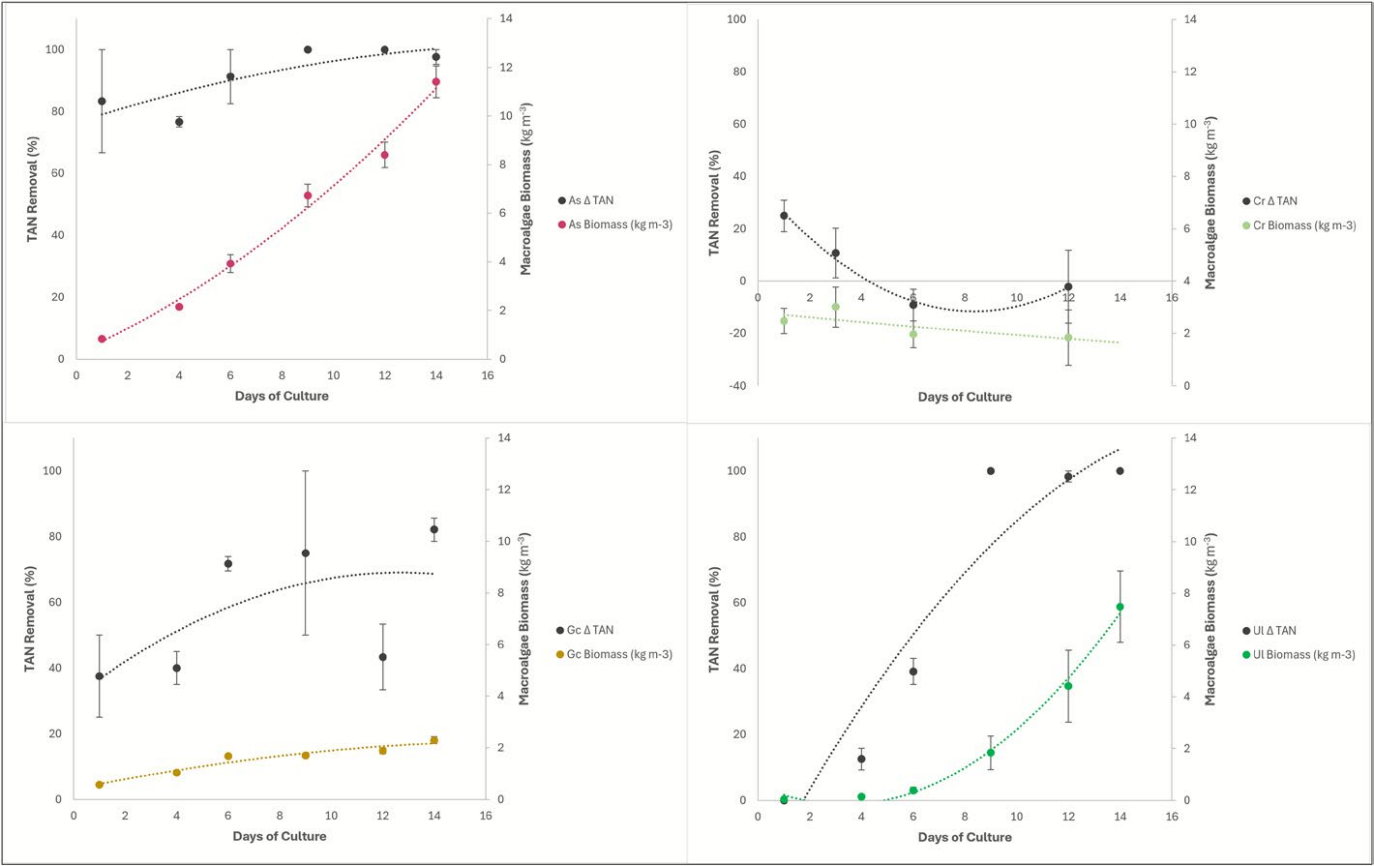
Δ TAN percentage of the macroalgae effluent water for each macroalgae species plotted against the biomass (g) of each macroalgae through the length of the trial. Top left is *Agardhiella subulata*, top right is *Caulerpa racemosa*, bottom left is *Gracilaria caudata*, bottom right is *Ulva lactuca*

Water quality data showed that in all species of macroalgae tested, there were significantly reduced TAN concentrations of the fish effluent water following passage through the macroalgae culture tanks. At the flow rates and HRTs utilized in this study, it took *Agardhiella subulata* 8 days to increase biomass enough to bring the TAN concentration below a detectable limit of 0.01 mg L^−1^ (henceforth referred to as zero mg L^−1^), *Gracilaria caudata* 11 days, and *Ulva lactuca* 9 days, while *Caulerpa racemosa* did not grow enough to lower the TAN levels to 0 throughout the 2-week trial. Corresponding macroalgae biomass densities at the point at which TAN concentrations in effluent water were virtually eliminated are as follows: *Agardhiella subulata* removed 100% of TAN from the fish effluent when it reached 6.73 kg m^−3^, *Gracilaria caudata* removed 82% of the TAN from the fish effluent when it reached 2.29 kg m^−3^, and at 1.83 kg m^−3^ *Ulva lactuca* removed 100% of the TAN from the fish effluent; while *Caulerpa racemosa* densities were not high enough to remove much of the TAN from the fish effluent water maxing out at 10% removal before it had an apparent die off. Figure 3 shows the relationship of the macroalgae biomass and the percent (%) TAN removed by the macroalgae. *Ulva lactuca*, *Agardhiella subulata*, and *Caulerpa racemosa* biomass have an inverse relationship with TAN percent left in the water, while *Gracilaria caudata* does bring TAN percentage down but does not display a clear relationship (Fig. 3).

The ANCOVA analysis on this data showed that TAN concentration in the macroalgae tank water depends on both the day of the trial and macroalgae species, and the interaction between the species and trial duration is important in the TAN levels as well (*p*-value: 0.0169). There were significant *p*-values when comparing all four macroalgae’s TAN uptake to each other (*p*-value: 1.65E − 11) and their uptake over time (*p*-value: 6.27E − 05).

Additionally, the ANCOVA analysis comparing each macroalgae species to the fish effluent showed varying significance between macroalgae and their ability to reduce nutrients from the fish effluent. *Agardhiella subulata* algae effluent TAN concentration was significantly different from the fish effluent TAN concentration regardless of time (*p*-value: 7.05E − 07). *Caulerpa racemosa* significantly decreased the TAN concentration from the fish effluent regardless of time (*p*-value: 0.000857). *Gracilaria caudata* significantly decreased the TAN concentration from the fish effluent regardless of time (*p*-value: 0.00147). Finally, the *Ulva lactuca* water TAN concentration was significantly different from the fish effluent TAN concentration regardless of time (*p*-value: 0.00119). Equations of lines depicted in Fig. 3 are detailed in the Supplementary Data file that accompanies this manuscript.

#### Phosphate

Water testing for phosphate showed that none of the macroalgae species was able to lower the phosphate to below the detection limit. The ANCOVA analysis on this data showed that the phosphate concentration in the macroalgae tank water depends on both time and macroalgae species, and the interaction between the species and time is important to the phosphate concentrations as well (*p*-value: 0.000784). There were significant differences in phosphate uptake between the macroalgae species (*p*-value: 1.09E − 07), and over time (*p*-value: 9.24E − 13).

**Fig. 4:**
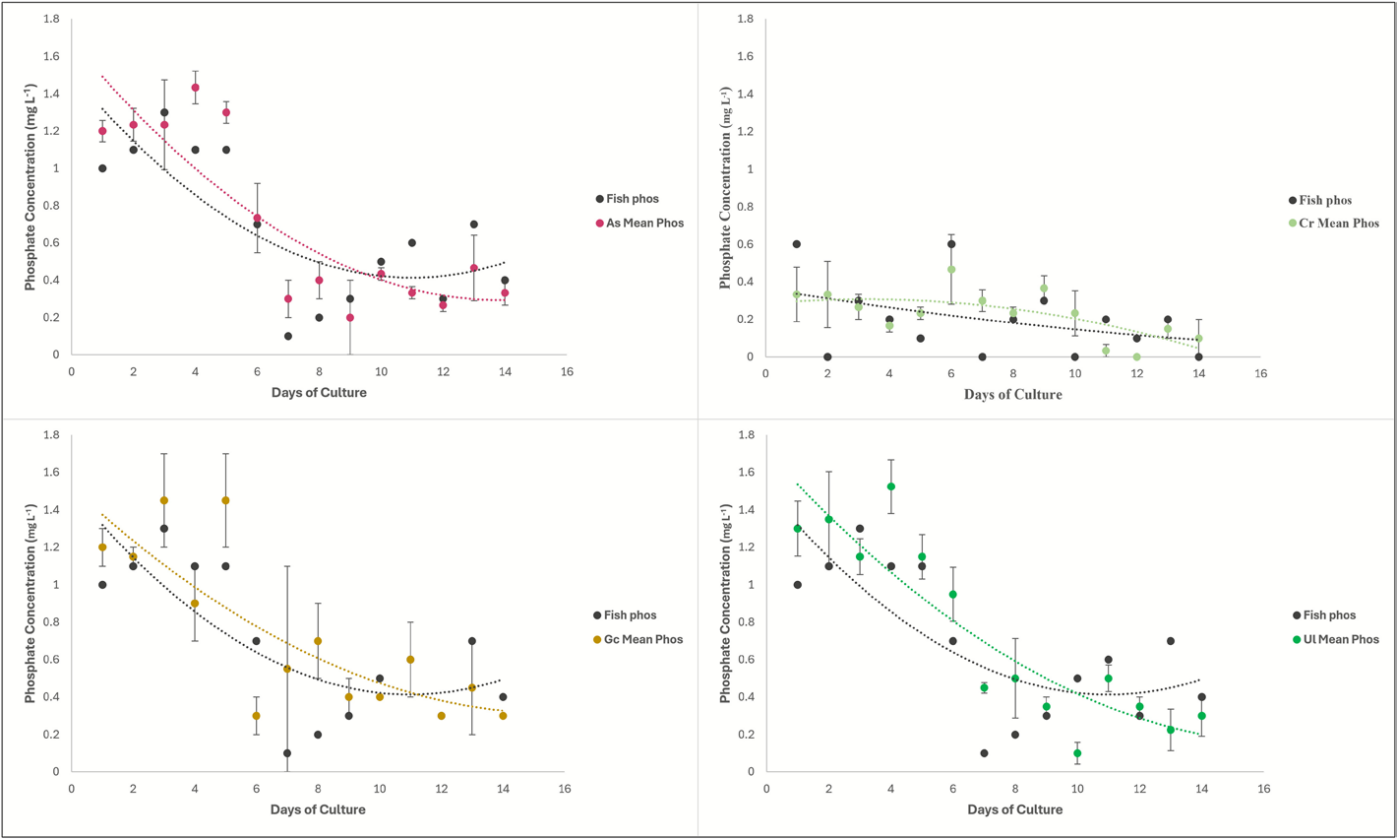
Graphs of the phosphate concentrations in the outflow of each macroalgae species compared to the fish effluent phosphate levels. Top left is *Agardhiella subulata*, top right is *Caulerpa racemosa*, bottom left is *Gracilaria caudata*, bottom right is *Ulva lactuca*

The ANCOVA comparing each macroalgae species to the fish effluent showed no significant difference between any of the four species of macroalgae and the fish effluent (Fig. 4). Equations of lines depicted in Fig. 4 are detailed in the Supplementary Data file that accompanies this manuscript.

#### pH

**Fig. 5:**
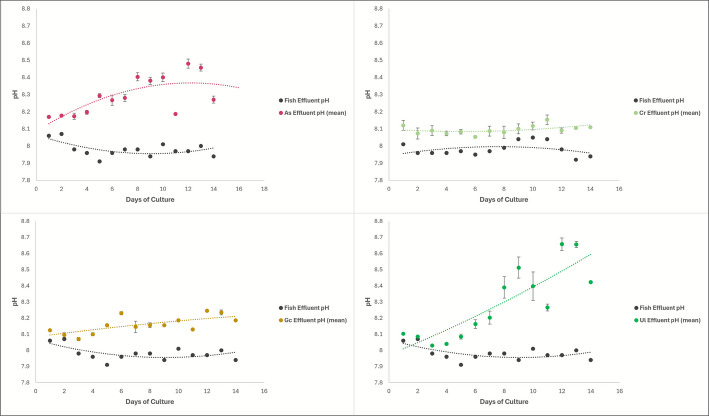
Graphs of the pH of the macroalgae effluent compared to the fish effluent pH. Top left is *Agardhiella subulata*, top right is *Caulerpa racemosa*, bottom left is *Gracilaria caudata*, bottom right is *Ulva lactuca*

All the tested macroalgae species significantly increased pH compared to the fish effluent water. Water quality data showed that the average pH for all fish effluent water samples was 7.98 ± 0.008, while the average pH of water following passage through the macroalgae tanks was 8.20 ± 0.200 (Fig. 5).

The ANCOVA of this data showed that the pH in the macroalgae tank water depends on both time and macroalgae species, and the interaction between the species and time is important in the pH as well (*p*-value: 1.20E − 06). There were significant *p*-values when comparing all four macroalgae tanks’ pH to each other regardless of time (*p*-value: 2.68E − 09), and their uptake was significantly different over time (*p*-value: 3.60E − 09).

Additionally, the ANCOVA comparing each macroalgae species to the fish effluent showed varying significance between macroalgae (Fig. 5). *Agardhiella subulata* and *Ulva lactuca* had significant differences in pH from the fish effluent, regardless of time (*p*-value: 5.81E − 12 and 3.37E − 09, respectively). Additionally, *Agardhiella subulata* and *Ulva lactuca* tank water pH significantly changed over time (*p*-value: 0.00185 and 4.76E − 06, respectively), while *Caulerpa racemosa* and *Gracilaria caudata* only had significant differences in pH from the fish effluent regardless of time (*p*-value: 2.61E − 08 and 3.30E − 11, respectively). Equations of lines depicted in Fig. 5 are detailed in the Supplementary Data file that accompanies this manuscript.

#### CO₂

The calculated CO₂ concentrations, in the form of dissolved CO₂, for every macroalgae tank effluent and the fish effluent indicated that, at all points in the trial and in all species of macroalgae, the dissolved CO₂ in the macroalgae tank water was lower than that of the fish effluent water. Additionally, it showed that effluent from the *Ulva lactuca* tanks had the lowest dissolved CO₂ concentration out of the four species of macroalgae.

The ANCOVA analysis on this data showed that dissolved CO₂ concentration in the macroalgae tank water depends on both time and macroalgae species, and the interaction between the species and time is important in the CO₂ concentrations as well (*p*-value: 4.58E − 06). There were significant *p*-values when comparing the CO₂ uptake of all four macroalgae to each other (*p*-value: 1.34E − 08) and their uptake over time (*p*-value: 1.37E − 10).

**Fig. 6:**
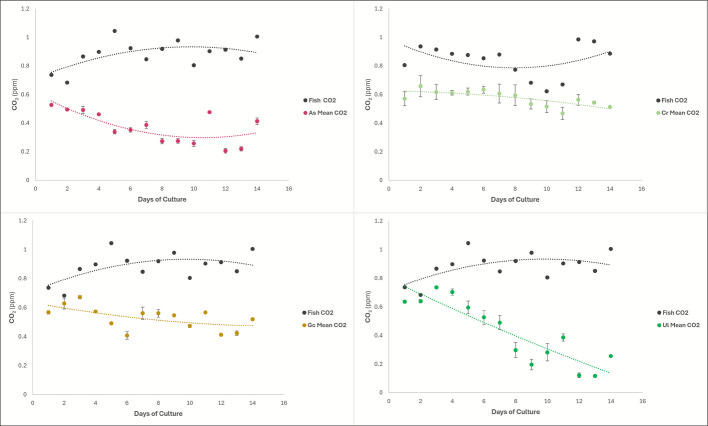
Graphs of the CO₂ concentrations of each macroalgae species’ effluent water compared to the fish effluent CO₂ concentration. Top left is *Agardhiella subulata*, top right is *Caulerpa racemosa*, bottom left is *Gracilaria caudata*, bottom right is *Ulva lactuca*

Additionally, the ANCOVA comparing each macroalgae species to the fish effluent showed varying significance between macroalgae (Fig. 6). The *Agardhiella subulata* tank CO₂ concentration was significantly different from the fish effluent CO₂ concentration regardless of time and was significantly different over time (*p*-value: 7.87E − 14 and 0.00301, respectively). *Caulerpa racemosa* significantly decreased the CO₂ concentration from the fish effluent regardless of time (*p*-value: 1.59E − 07). *Gracilaria caudata* significantly decrease the CO₂ concentration from the fish effluent, regardless of time and over time (*p*-value: 1.92E − 11 and 0.00863, respectively). Finally, the *Ulva lactuca* tank CO₂ concentration was significantly different from the fish effluent CO₂ concentration regardless of time, and the two were significantly different over time (*p*-value: 0.000514, 4.13E − 12, and 1.35E − 06, respectively). Equations of lines depicted in Fig. 6 are detailed in the Supplementary Data file that accompanies this manuscript.

#### Dissolved oxygen (DO)

Water quality data showed that regardless of species, the dissolved oxygen in the algae tanks was most often lower than that in the fish effluent. There were significant differences in DO of the macroalgae effluent between macroalgae species (*p*-value: 4.17E–11). Additionally, the DO increased significantly in the macroalgae effluent over time (*p*-value: 6.68E–15).

#### Alkalinity

The alkalinity of the macroalgae effluent was not significantly different from the alkalinity of the fish effluent in any macroalgae species. The ANCOVA comparing the macroalgae species’ alkalinities to each other showed significant *p*-values (*p*-value: 1.30E − 06) and a significant increase over time (*p*-value: 5.24E − 07).

### Growth

**Fig. 7:**
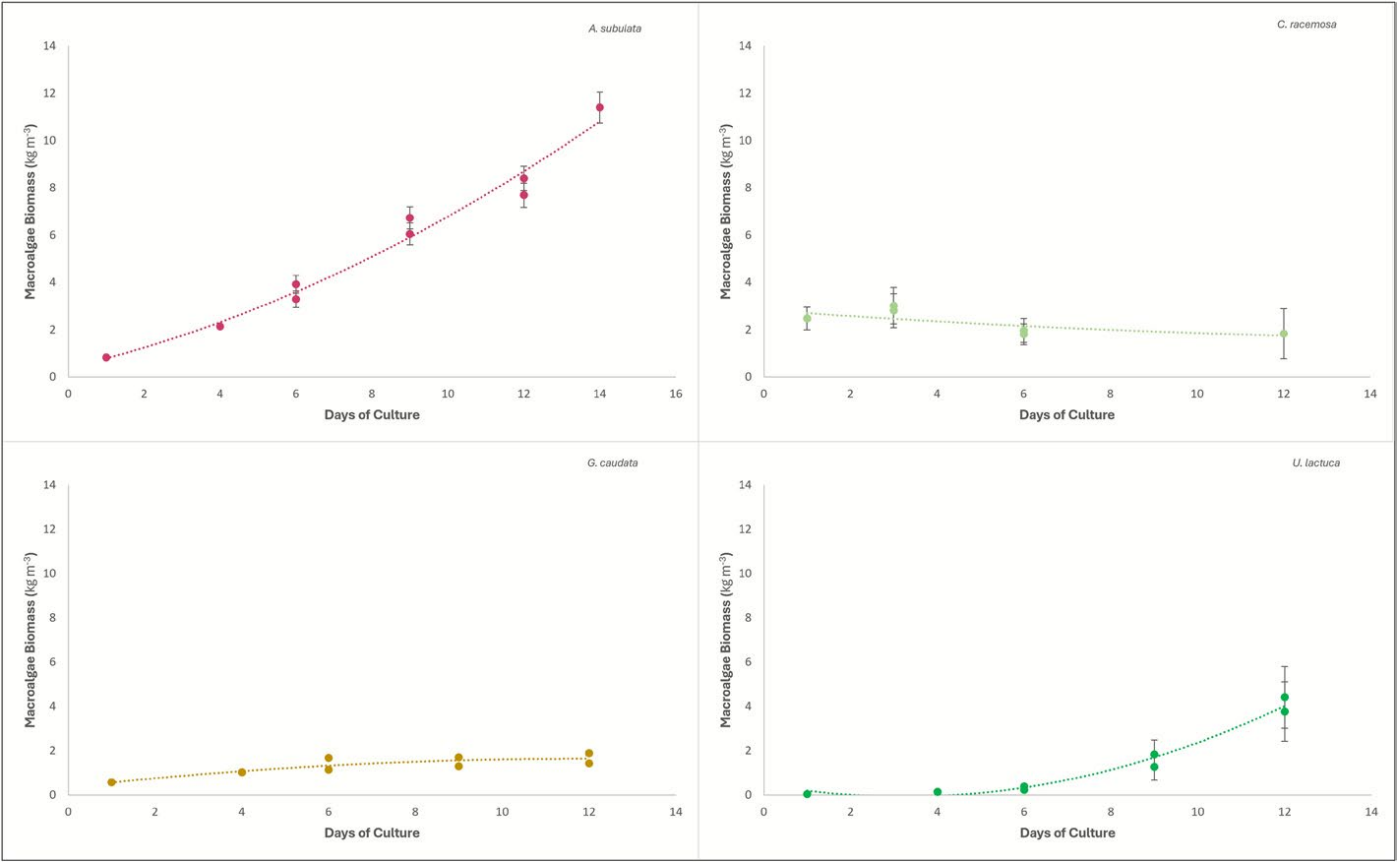
Growth curves displayed in macroalgae biomass kg m^-^^3^ (180 L tank) of *Agardhiella subulata* (top left), *Caulerpa racemosa* (top right), *Gracilaria caudata* (bottom left), and *Ulva lactuca* (bottom right), including the periodic sharp drops in weight when sample material was collected and removed from culture tanks

The growth data showed that *Ulva lactuca* exhibited exponential growth. From day 6 to 9, the *Ulva lactuca* increased 721% in wet weight and overall went from 2 g (0.011 kg m^−3^) wet weight to 1347 g (7.48 kg m^−3^) wet weight (Fig. 7). *Agardhiella subulata* had consistent growth with the highest total weight at the end of the trial. *Agardhiella subulata* started at 150 g (0.83 kg m^−3^) and ended at 2052 g (11.40 kg m^−3^) wet weight (Fig. 7). *Gracilaria caudata* had an overall increase in wet weight, but it was much smaller than that of *Ulva lactuca* or *Agardhiella subulata*, with a starting wet weight of 103 g (0.57 kg m^−3^) and a final wet weight of 413 g (2.29 kg m^−3^) (Fig. 7). *Caulerpa racemosa* demonstrated a different growth pattern involving turnover of old growth versus new growth, and this resulted in a somewhat opposite relationship with growth where over time, the *Caulerpa racemosa* got lighter in wet weight, starting at an average of 446 g (2.48 kg m^−3^) and ending with an average wet weight of 330 g (1.83 kg m^−3^) (Fig. 7).

In addition to growth, wet weight was used to calculate the water percentage in the macroalgae after drying. *Agardhiella subulata* had a wet weight (WW) of 1071.843 g and dried down to 90.213 g, giving a percent water of 88.12%. *Caulerpa racemosa* has a WW of 211.007 g and dried to 21.657 g, yielding a percent water of 90.266%. *Gracilaria caudata* had a WW of 341.858 g and dried to 46.551 g, giving a percent water of 92.66%. Finally, *Ulva lactuca* had a WW of 1226.249 g and dried down to 137.706 g, yielding a percent water of 91.10%. Based on this information, *Gracilaria caudata* had the highest water content of all four species. Equations of lines depicted in Fig. 7 are detailed in the Supplementary Data file that accompanies this manuscript.

### Elemental analysis

**Fig. 8:**
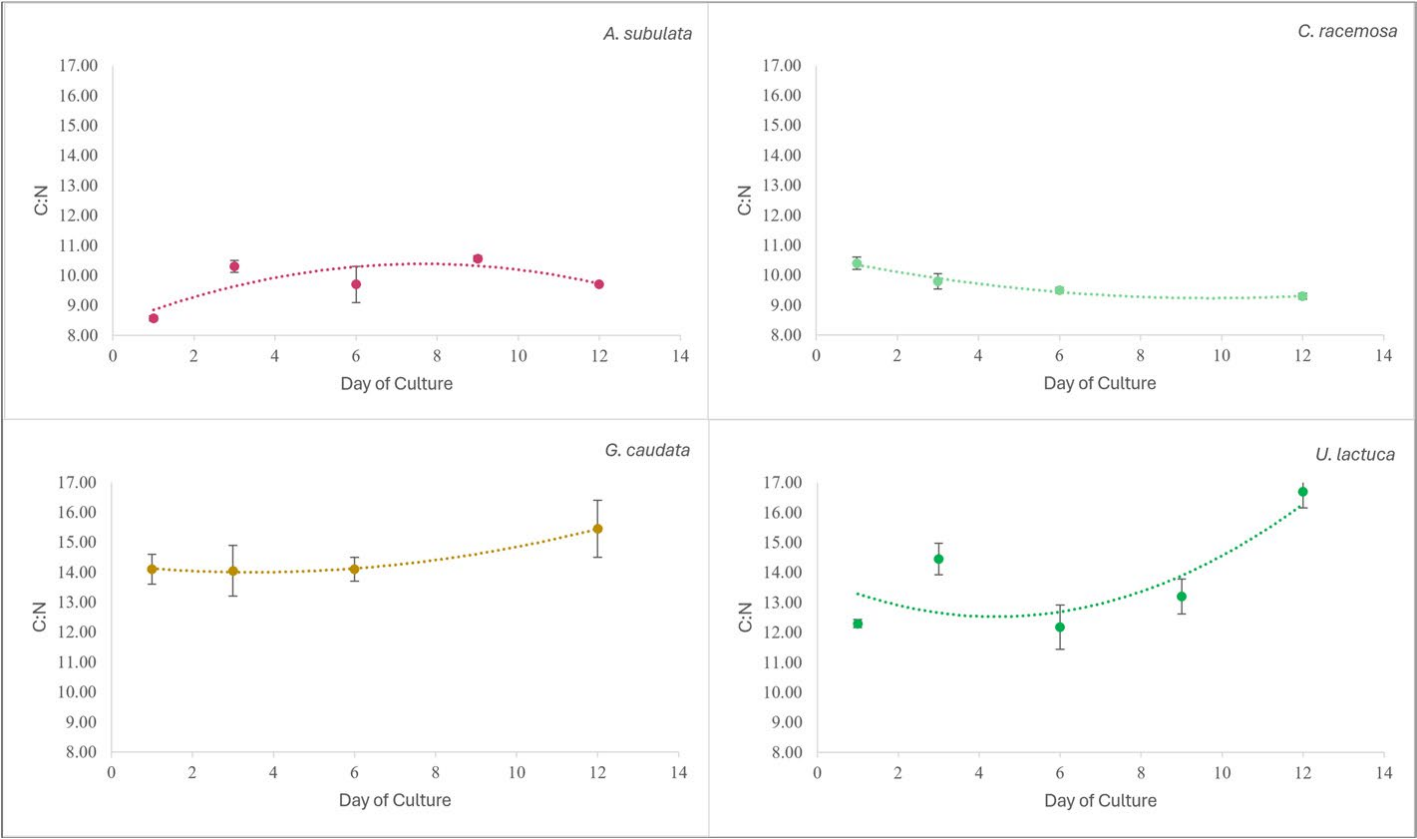
Graph of carbon-to-nitrogen ratio over time. Top left is *Agardhiella subulata*, top right is *Caulerpa racemosa*, bottom left is *Gracilaria caudata*, bottom right is *Ulva lactuca*

The elemental analysis showed varying significance of the carbon:nitrogen (C:N) ratio in the analysis of covariance. There was a significant difference in the C:N ratio between macroalgae species (*p*-value: 4.93E − 05). However, there was no significant difference in the C:N ratio over time, and no significant interaction effect between time and species’ C:N ratios. The C:N ratio was highest in *Ulva lactuca* (Fig. 8), and the second highest in *Gracilaria caudata* (Fig. 8), while *Agardhiella subulata* and *Caulerpa racemosa* had lower C:N ratios, indicating uptake of less carbon alongside the nitrogen they took up (Fig. 8). Equations of lines depicted in Fig. 8 are detailed in the Supplementary Data file that accompanies this manuscript.

## Discussion

This study provides some of the first comparative assessments of native macroalgae species of the Southeast, Gulf of Mexico, and Caribbean regions that have been proposed as candidate macroalgae species in IMTA production systems. With significant interest in further development of marine aquaculture throughout these regions, the findings in this study provide insights that can be utilized to guide the selection of extractive macroalgae species for use in marine IMTA operations culturing marine finfish in the regions. Results of this work demonstrate that nearly all the compounds of concern, from a marine aquaculture fed-species effluent standpoint, can be significantly reduced—and in many cases, eliminated—through the cultivation of some of the species of macroalgae tested in this study. Such results demonstrate the potential of IMTA in the Southeast, Gulf of Mexico, and Caribbean regions and offer potential mitigation solutions for many of the most prominent social license concerns regarding the development of marine aquaculture operations for fed-species. The primary aim of this study was to observe the nutrient assimilation abilities, nutrient content, growth, and elemental content of four algal species. This was done to provide an understanding of which macroalgae species from the Southeast and Gulf of Mexico regions perform the best in these different categories, thus providing stakeholders with a guide to select a desirable species of macroalgae to utilize and implement in their operations.

### Nutrient content

Whether for human consumption or animal nutrition studies, protein composition is of great importance for the market value of seaweed. In nearly all species assessed in this study, the macroalgae cultured in the IMTA system was fortified with nutrients compared with previously reported compositional analyses of such species. Studies show that seaweed cultured in IMTA systems showed higher protein content than wild seaweed (Zhu et al. 2025). Several studies show the wild *Caulerpa racemosa* populations can have a protein content with a range between 0.6 and 20%, while cultured *Caulerpa racemosa* can have upwards of 20% protein (de Gaillande et al. 2017; Hao et al. 2019; Magdugo et al. 2020; Peñalver et al. 2020). This study found *Caulerpa racemosa* to have 25% crude protein content at the end of the 2-week trial, which is higher than the protein content found in the studies mentioned previously (Table 1). *Agardhiella subulata* had an end protein content of 22.66%, which was the second highest in this study (Table 1). Of the small amount of *Agardhiella subulata* research, Rawiwan et al. (2022) found the protein content of the Rhodophyceae class to be within a range of 2.7–47%, aligning with the findings of this study’s *Agardhiella subulata*. *Gracilaria* is also known to have a large range for protein content (5–37%), and the *Gracilaria caudata* in this study had a protein content of 21.22% (Table 1) (Abreu et al. 2011; Holdt and Kraan 2011; Lozano et al. 2016). Wild cultured *Ulva lactuca* is known to have 5–12% protein content, and the *Ulva lactuca* from this study had 13.56% (Table 1) (Magdugo et al. 2020; Shuuluka et al. 2013). While the *Ulva lactuca* from this study had protein comparable to its wild counterparts, it did not model the protein content found in other IMTA-rendered *Ulva* studies, which showed upwards of 44% protein (Ben-Ari et al. 2014; Shpigel et al. 2017). This may indicate that the conditions of this study did not provide *Ulva lactuca* with as much nutrients as it can take up. This is reflected in the exponential growth of *Ulva lactuca* and its tank size possibly being a limiting factor, and its ability to bring the TAN down to 0 in its tank. This suggests that *Ulva lactuca* could potentially be grown to a point of having a higher protein content if more nitrogen-based nutrients were provided.

A further breakdown of protein into amino acids is important to know for a better understanding of the nutritional aspect macroalgae brings to aquaculture research. Of the essential amino acids finfish require in their diets, these macroalgae contained all but two: methionine and tryptophan (Fig. 2) (Trushenski et al. 2006). This indicates that if these species of macroalgae were to be used in a finfish diet, there would need to be a supplementation with methionine and tryptophan to meet finfish’ nutritional requirements.

The total carbohydrate content was calculated as the difference between total weight and the provided moisture, protein, ash, and fat percentages. *Ulva lactuca* contained the highest total carbohydrate percentage (Table 1). The importance of this is explained further in the “Elemental analysis” section. A study done on red algae found that the production of carrageenan, a carbohydrate, was lower when the growth rate was high (Freile-Pelegrin and Robledo 2006). This could indicate why the red algae species in this study, such as *Agardhiella subulata*, had lower carbohydrates than *Gracilaria caudata*, because *Agardhiella subulata*’s growth was much faster (Fig. 7).

The final item of importance that was compositionally analyzed was the fatty acid profile. The *Agardhiella subulata* omega-3 was 0.27% and omega-6 was 0.36% of the dry weight (Table 2). For perspective in human nutrition, the fatty acid data from this study suggests that human consumption of 90 g dry weight of *Agardhiella subulata*, 293 mg of omega-3, and 324 mg of omega-6 would be consumed. The average fish oil human nutraceutical pill is about 300 mg of omega-3 and omega-6 combined (National Institute of Health 2023). This indicates that just half of the 90 g dry weight sample of *A. subulata*, i.e., 45 g dry weight, provides the equivalent omega-3 and omega-6 amounts as an average commercially available fish oil human nutraceutical pill. *Caulerpa racemosa* had an even higher omega-3 sample basis percent of 0.49%, and *Gracilaria caudata* had a higher omega-6 sample basis percent of 0.46%, but neither of these had the high combined omega-3 and omega-6 profile that *Agardhiella* did (Table 2). Therefore, using the average commercially available 300 mg fish oil human nutraceutical pill as a baseline, the macroalgae that provide the highest amounts of heart-healthy lipids would be *Agardhiella subulata* and *Caulerpa racemosa* with a consumption amount of 45 g and 42 g DW, respectively.

### Minerals and metals

Due to the ability of macroalgae to uptake and/or adsorb minerals and metals directly from the water column, calcium, phosphate, magnesium, iron, potassium, arsenic, lead, mercury, and cadmium were analyzed for each species in this study. The results report the dry weight ppm of minerals and metals (Table 1). The U.S. Food and Drug Administration (FDA) currently classifies unprocessed seaweed as a “raw agricultural commodity” (RAC) and therefore does not regulate it as a food product (National Sea Grant Law Center 2023). However, for the purposes of this study, we provide comparative assessments of selected seaweed compositional elements based off recommended levels for humans from other food items and supplements (FAO 2022; FDA, 2022; National Institute of Health 2022a, b, 2023a, b, 2024). The FDA recommends that under 1 ppm of mercury is safe for human consumption, and all four species had lower than 0.05 ppm dry weight mercury, indicating they are safe for human consumption (Table 1) (U.S. Food and Drug Administration 2024a). This is not unusual, as seaweed, a primary producer, is at the base of the food web, so mercury is less likely to accumulate. All four species of macroalgae also had cadmium levels that were below FDA limits. The FDA recommends no more than 0.4 ppm cadmium, and the highest cadmium level was 0.16 ppm dry weight in *Agardhiella subulata* (Table 1) (U.S. Food and Drug Administration 2024b). Lead levels accepted by the FDA for food and supplements are 0.05 ppm (U.S. Food and Drug Administration 2024c). Therefore, the only macroalgae in this study that are outside of that range are *Caulerpa racemosa* with a lead content of 0.7 ppm dry weight. In terms of arsenic, results from the present study align with some of the recent findings for sugar kelp (*Saccharina latissima*) from both wild harvest and aquaculture-produced sources in the Northeast U.S. (Shaughnessy et al. 2023) and past work with *Gracilaria tikvahiae* and *Saccharina latissima* in Long Island Sound and New York estuaries (Kim et al. 2019b), in that all species of macroalgae in the present study had measurable levels of inorganic arsenic (Table 1). *Agardhiella subulata* had an average arsenic concentration of 8 ppm, *Caulerpa racemosa* had 11.4 ppm, *Gracilaria caudata* had 7.3 ppm, and *Ulva lactuca* had 4.8 ppm, all of which represent dry weight concentrations. Environmental concentrations of arsenic are a topic of increasing concern globally, and this could be a potential cause of arsenic levels seen in macroalgae in this study. Studies show that Biscayne Bay has, in the past, shown elevated levels of arsenic, with one study finding 0.3 ppm inorganic arsenic in Biscayne Bay sediment, and another finding inorganic arsenic to exceed threshold effect levels in 44% of sediment samples (Fernandez, 2004; Long et al. 1999). However, any insights on potential human health impacts associated with food products containing measurable levels of total arsenic need to consider the important role of arsenic speciation in terms of varying toxicity of arsenic species that may be present within total arsenic concentration data (Monagail and Morrison 2019), though such analyses were beyond the scope of the present study. Based on the findings of the present study, *Caulerpa racemosa* had the highest levels of metals of the four macroalgae species tested, though such findings could be an artifact of the more recent wild-stock collection of *Caulerpa racemosa* used in this study and may not be an accurate reflection of this species in general once cultured in captivity over extended periods of time. In terms of human consumption of seaweed species, the ability of seaweed to adsorb trace elements from seawater is part of the reason seaweed can be very nutritious, yet there can also be the potential for unwanted levels of various metals in seaweeds. This has led to some commercial suppliers of seaweed products applying labels, such as California Prop 65 labels, to seaweed products that may be destined for human consumption.

Referring to the “Minerals and metals” section, of the minerals the macroalgae took up; iron was highest in *Caulerpa racemosa*, with 66.2 ppm, which is also 1.38 mg of iron in the 21 g DW sent for analysis (Table 1). This means that the average man (age 19 +) could eat 125 g of the *Caulerpa racemosa* from this study and get the daily recommended intake of iron (8 mg for men, 18 mg for women) (National Institute of Health 2023). This is consistent with a previous study done on *Caulerpa racemosa*, stating that there can be up to 81 mg of iron in 100 g of *Caulerpa racemosa* (de Gaillande et al. 2017). A study done by Leandro et al. (2019) found that consuming 10 g of *Ulva lactuca* could provide 70% of the daily magnesium requirement. This study found 3600 mg magnesium in 100 g of *Ulva lactuca*, which is consistent with the findings in Leandro et al. (2019). Another study found that consuming 15 g WW of *Caulerpa racemosa* could give you your recommended daily intake of calcium (de Gaillande et al. 2017). While the *Caulerpa racemosa* in this study did contain a good amount of calcium, 100 g DW of the *Caulerpa racemosa* from this study would only provide half the recommended daily intake of calcium (National Institute of Health 2024).

### Water quality

During photosynthesis, seaweeds take up CO₂ and produce oxygen. However, when looking at the DO data from this study, it shows lower DO in all algal tanks from the fish effluent. This was likely because the fish tank was supplied with supplemental oxygen to maintain the commercial stocking densities used in this trial, while the macroalgae tanks were aerated. Off-gassing and utilization of oxygen as part of the growth process in the macroalgae likely contributed to differences noted in DO concentrations between fish culture and macroalgae tanks.

This study found that all four macroalgae species were able to lower the total ammonia nitrogen concentration and CO₂ of the fish effluent water significantly, some even lowering the TAN down to undetectable concentrations using the methods in this study (Fig. 3). This provides promising information that land-based and offshore IMTA systems may bring the nutrient load from fish effluent down to a safe level. *Agardhiella subulata* had the most significant *p*-value of all species when comparing the TAN levels in the macroalgae tank to the fish effluent (*p*-value: 7.05E − 07) (Fig. 3). This indicates that *Agardhiella subulata* might be the best choice for nitrogen uptake in an IMTA system, with *Ulva lactuca* in close second.

The pH in the algal tanks was always significantly higher than the fish effluent water (Fig. 5). This is likely due to the time samples were taken, allowing for significant photosynthesis to occur before samples were taken, but also due to the equilibration of CO_2_ with the overlying atmosphere. As part of the photosynthetic process in macroalgae, CO_2_ is taken up, and this process raises the surrounding pH while also lowering the CO₂ concentrations in the water column (Gerardi et al. 2015).

Accordingly, CO_2_ concentrations in the outflow of the macroalgae tanks were, in all cases, lower than the fish effluent. As with changes in oxygen concentration, part of this change in CO_2_ concentration resulted from equilibration with the overlying atmosphere; however, the difference in CO_2_ concentration between the outflow of the different macroalgae species suggests that some species were more effective than others at removing CO_2_ via photosynthesis. The algae with the most significant *p*-value when assessing the CO₂ consumption from the fish effluent was *Agardhiella subulata* with a *p*-value of 7.87E − 14, and *Ulva lactuca* came in a close second with a *p*-value of 4.13E − 12 (Fig. 6). This suggests that either of these macroalgae would be a good choice when looking for a seaweed capable of up taking CO₂ from an IMTA system. Studies have shown areas where macroalgae are grown to be carbon sinks, which support the findings of CO_2_ concentration decrease in this study (Zhu et al. 2025). This is also important for the blue economy; two of the Southeast U.S./Caribbean/Gulf of Mexico native species in this study were capable of significantly decreasing the CO₂ content of the water, making these seaweeds more desirable to farmers, not just for bio-mitigative reasons but also when looking at the climate crisis and the possibility of carbon credits.

The algal tanks had significantly different phosphate levels when compared to each other, but when compared to the fish effluent, they were unable to significantly change the phosphate levels. This is not entirely surprising due to the relationship between nitrogen uptake and phosphate uptake. Since the macroalgae could lower the nitrogen in the water below detection, their growth was likely nitrogen-limited, thus leaving excess unused phosphate (Perini and Bracken 2014; Saito et al. 2008). *Caulerpa racemosa* had the lowest phosphate concentrations in tank outflow of all the macroalgae throughout the trial (Fig. 4). This is likely due to its relatively high phosphate requirements at optimal growth rates (N:P 6:1; Harwanto et al. 2020). The phosphate concentrations of both the fish effluent and the algae tanks did not exceed 1.6 mg L^−1^, and although the phosphate did not decrease significantly, it did decrease overall in the 2-week trial (Fig. 4).

The CO₂ concentration, pH, and alkalinity all provide promising information regarding the climate change conversation. The CO₂ drawdown in this study, the pH increase due to CO₂ drawdown, and a steady alkalinity show a strong ability to buffer the system and keep pH steady and not acidic, demonstrating promise in IMTA practices helping to mitigate ocean acidification.

One area with room for improvement would be to have gotten water quality parameters of the facility’s water before entering the yellowtail snapper tank. The water to the facility is pulled from Biscayne Bay into settling tanks, and from the settling tanks goes through mechanical filtration of sand filters and through a UV unit. Testing this clean water coming into the facility and thus into the yellowtail snapper tank would have helped us understand how much of the nutrient load going to the seaweed tanks was contributed by the fish.

### Growth

The growth for each species in this study was predicted to be positive because the algal tanks were in natural sunlight for optimal photosynthesis, and they were provided a large amount of nutrients from the yellowtail snapper upstream in the IMTA system. While *Agardhiella subulata*, *Gracilaria caudata*, and *Ulva lactuca* all grew over the trial duration, *Caulerpa racemosa* did not (Fig. 7).

*Caulerpa racemosa* may have struggled due to an excess of sunlight. When the *Caulerpa racemosa* was put into the system to acclimate, it was put into three tanks at approximately 0.0017 kg m^−3^ each. After a few days, the *Caulerpa racemosa* was yellowing in the spots of the tank with the most light, indicating a need for greater shading of the tank. Following the addition of shade cloth to help limit the amount of light, the *Caulerpa racemosa* rebounded. However, the initial period of intense light could have caused some *Caulerpa racemosa* to die following initiation of the growth trial. Other potential explanatory factors in the lack of *Caulerpa racemosa* growth could be related to stocking density. The low stocking density could have caused the *Caulerpa racemosa* to die as well for a couple of reasons: high light because there was so much open white tank to reflect sunlight or too much water volume of nutrients for the *Caulerpa racemosa* to uptake, so there was other algal growth that might have killed off some sections of *Caulerpa racemosa*. One study found that with an increase in uptake of TAN, there is an increase in growth in *Caulerpa racemosa* (Harwanto et al. 2020). This could be an indication of the poor growth the *Caulerpa racemosa* exhibited in this study, because the *Caulerpa racemosa* was the least successful at up taking nitrogen, never significantly lowering TAN from the fish effluent. However, the more likely cause for the declining weight of the *Caulerpa racemosa* is that it did not have enough time to acclimate to its new system. Since the *Caulerpa racemosa* was wild-caught, it already contained properties it gained from its ocean environment. When the *Caulerpa racemosa* was introduced into this study system, and growth of the macroalgae was detected, the trial began. What may have happened is that the new growth on the macroalgae indicated positive growth at first, but the old stems of the *Caulerpa racemosa* did not do as well in this new system as the new growth did, so the old stem die-off was larger than the new growth in this system, giving the negative slope of wet weight. The best solution to this would have been to let the *Caulerpa racemosa* acclimate to its new system for longer, long enough that the old growth could die off and only new growth be involved in the trial.

The growth of the other species was consistent with previous studies. A study done by Zhou et al. on *Gracilaria* spp. growing near fish farms found a maximum growth rate of 11% per day (Zhou et al. 2006). This is consistent with the findings of growth of *Gracilaria caudata* in this study with a maximum growth rate of 27% per day (Fig. 7). This study found slightly higher growth rates likely due to having a direct fish effluent source rather than just being grown near a fish farm; additionally, these macroalgae had very low epiphytes to begin with and were in a tumbling culture. Another study done on the potential of green algae in aquaculture states the high growth rates and low epiphytic growth of *Ulva* species, which is exemplified by the high growth rates and lack of epiphytic algal growth on the *Ulva lactuca* in this study (Moreira et al. 2022). Another positive finding from this study was that *Ulva lactuca* had the least epiphytes of all the macroalgae species.

A study using *Agardhiella subulata* by Lohroff et al. (2021) found that *Agardhiella subulata* peaked at day 9 of their trial with 17 kg m^−3^ in a 235-L tank, and growth decreased after that point due to self-shading impacts associated with the high biomass density of macroalgae. Such findings indicate that the carrying capacity of the 235-L tanks utilized in the Lohroff et al. (2021) study was reached, while in the present study, the *Agardhiella subulata* in 180-L tanks did not reach a point of decreasing growth rate over the 2-week study period even at the final harvest density of 11 kg m^−3^ (Fig. 7).

### Elemental analysis

The carbon and nitrogen ratio (C:N) from throughout the trial can give insight into the protein, carbohydrate, and fat content of the macroalgae. A higher C:N indicates more carbon-rich organic components, while a lower C:N indicates more nitrogen-rich components. The N-rich component looked at in this study was protein, while the C-rich components were carbohydrates and lipids/fats. If the end points of the organic composition data make sense next to the C:N ratio endpoints from the elemental analysis, then it can suggest that the C:N ratio is reliable for estimating the protein and carbohydrate/lipid content change over time in the study. The C:N ratio was lower in *Agardhiella subulata* and *Caulerpa racemosa*, which correlates with the organic component data (Fig. 8). *Agardhiella subulata* and *Caulerpa racemosa* both had the highest protein content, and protein is a nitrogen-rich component; therefore, this elemental data reflects that finding. Additionally, *Ulva lactuca* had the highest C:N ratio and best CO₂ uptake, indicating that *Ulva lactuca* should have lower nitrogen-rich components and more carbon-rich components, which is accurate to the nutrient content data. The *Ulva lactuca* in this study had the lowest protein and the highest carbohydrate, indicating more carbon-based components, which correlates with the high C:N ratio.

The carbon sequestration by these macroalgae, specifically *Ulva lactuca* and *Gracilaria caudata*, showed high C:N ratios, indicating a strong affinity for carbon uptake without needing to deplete the nitrogen and other nutrients useful to the ocean ecosystem (Fig. 8). This is one more finding from this study that supports seaweed’s important role in the climate change conversation. These macroalgae not only help with the CO₂ impact of a land-based farm but could potentially provide good carbon sequestration in open ocean farms because of the high C:N ratio. The *Ulva lactuca* and *Gracilaria caudata* having high C:N suggests their biomass could be manipulated to maximize carbon uptake while allowing for other nutrients to get through or be taken up as well.

The ability of *Ulva lactuca* and *Gracilaria caudata* to produce organic tissues with a high ratio of carbon to nitrogen indicates they may be a good choice when looking at ways to store carbon long term (Fig. 8). A study by Ross et al. (2023) suggested sinking seaweed into the deep sea to sequester their carbon long term. The findings from this project indicate the potential use of *Ulva lactuca* and/or *Gracilaria caudata* for sinking into the deep ocean for their carbon uptake abilities.

While a full isotopic mass balance was not attempted for the IMTA system, results of natural-abundance bulk isotope analysis of macroalgal tissue across several time points generally support the findings from biomass growth and nutrient drawdown for each species. Generally, δ^13^C and δ^15^N values of photosynthetic tissue reflect (1) the δ^13^C and δ^15^N values of the inorganic substrate from which the C (CO_2_) and N (ammonium or nitrate) were acquired and (2) isotopic fractionation during assimilation into organic structures, which can be affected by the rate of tissue growth relative to the supply of the inorganic substrates (Popp et al. 1998; Sigman and Casciotti 2001). Lack of new growth in *C. racemosa* is reflected in the δ^13^C and δ^15^N values of tissues that are different from other species and likely reflect the C and N sources in its original (wild) growth environment. With linearly increasing biomass during the experiment (Fig. 7), *A. subulata* grew at a constant rate, which is reflected in relatively constant δ^13^C and δ^15^N values of tissues across the four time points characterized. With a relatively low C:N ratio of tissue (~ 10; Fig. 8), *A. subulata* had a relatively high N requirement and thus began using fish effluent N early in the experiment, as reflected both in the TAN drawdown and the constant δ^15^N values over time. The constant growth rate of *G. caudata* (Fig. 7) is also reflected in the constant δ^13^C and δ^15^N values of its tissues over time, which were also similar to those of *A. subulata* and thus likely reflect the CO_2_ and N sources from the fish effluent. In contrast, *U. lactuta* experienced slow growth early in the experiment (Fig. 7) and had a high C:N ratio of tissue (~ 12–17; Fig. 8); as a result, the low δ^15^N values of its tissue and its low drawdown of TAN at early time points indicate that it was not taking up significant N from the fish effluent early in the experiment. At later time points, the biomass growth of *U. lactuta* was exponential, and the uptake of both C and N was evident from CO_2_ and TAN drawdown as well as the δ^13^C and δ^15^N values of its biomass, which approached the same values as *A. subulata* and *G. caudata*, again likely reflecting the uptake of CO_2_ and N from the fish effluent.

## Conclusions

This research provides industry-relevant insights and guidance for IMTA marine aquaculture in the Southeast U.S., Caribbean, and Gulf of Mexico regions. Table 3 summarizes which macroalgae species performed the best in each tested category. Such information allows stakeholders to assess what macroalgae species from the focal regions might be most beneficial to incorporate into marine IMTA production operations (Table 3). The information is separated into two base categories: active growth effects and end product nutrition, to aid in the choosing of a macroalgae based on the need for a biomitigative macroalgae or a market-savvy macroalgae.

**Table 3:** Stakeholder guide to which species of Southeast US/Gulf/Caribbean region native algae is best suited for a particular mitigative end goal

Parameter of interest	Best species	Good alternative species
Tan drawdown	*Agardhiella*	*Ulva*
Phosphate drawdown	*Caulerpa*	*Agardhiella*
CO₂ drawdown	*Ulva*	*Agardhiella*
O₂ production	*Ulva*	*Agardhiella*
pH increase	*Ulva*	*Agardhiella*
Protein	*Caulerpa*	*Agardhiella*
Total carbohydrates	*Ulva*	*Gracilaria*
Fiber	*Caulerpa*	*Ulva*
PUFA’s	*Agardhiella*	*Caulerpa*
Omega 6	*Gracilaria*	*Agardhiella*
Omega 3	*Caulerpa*	*Agardhiella*
Amino acids	*Caulerpa*	*Agardhiella*
Biomass	*Agardhiella*	*Ulva*
Calcium (Ca)	*Caulerpa*	*Agardhiella/Ulva*
Magnesium (Mg)	*Ulva*	*Agardhiella*
Iron (Fe)	*Caulerpa*	*Agardhiella*

## Supplementary Information

Below is the link to the electronic supplementary material.

Supplementary file1 (DOCX 16 kb)

## Data Availability

Data is provided within the manuscript or supplementary information files, and any further data is available upon request.

## References

[CR1] Abreu MH, Pereira R, Yarish C, Buschmann AH, Sousa-Pinto I (2011) IMTA with *Gracilaria vermiculophylla*: productivity and nutrient removal performance of the seaweed in a land-based pilot scale system. Aquaculture 312(1):77–87. 10.1016/j.aquaculture.2010.12.036

[CR2] Ben-Ari T, Neori A, Ben-Ezra D, Shauli L, Odintsov V, Shpigel M (2014) Management of *Ulva lactuca* as a biofilter of mariculture effluents in IMTA system. Aquaculture 434:493–498. 10.1016/j.aquaculture.2014.08.034

[CR3] Chopin T, Buschmann AH, Halling C, Troell M, Kautsky N, Neori A, Kraemer GP, Zertuche-González JA, Yarish C, Neefus C (2001) Integrating seaweeds into marine aquaculture systems: a key toward sustainability. J Phycol 37(6):975–986. 10.1046/j.1529-8817.2001.01137.x

[CR4] de Gaillande C, Payri C, Remoissenet G, Zubia M (2017) Caulerpa consumption, nutritional value and farming in the Indo-Pacific region. J Appl Phycol 29(5):2249–2266. 10.1007/s10811-016-0912-6

[CR5] FAO. (2022). The state of world fisheries and aquaculture 2022. 10.4060/cc0461en

[CR6] Fernandez, A. M. (2004). Distribution and occurrence of inorganic and organic contaminants in sediments and whole fish tissue of the Everglades and Biscayne National Parks [M.S., Florida International University]. 10.25148/etd.FI15101381

[CR7] Fleurence J, Morançais M, Dumay J, Decottignies P, Turpin V, Munier M, Garcia-Bueno N, Jaouen P (2012) What are the prospects for using seaweed in human nutrition and for marine animals raised through aquaculture? Trends Food Sci Technol 27(1):57–61. 10.1016/j.tifs.2012.03.004

[CR8] Food and Drug Administration. (2022, June). Fish and fishery products hazards and controls guidance. https://www.fda.gov/media/80637/download

[CR9] Freile-Pelegrín Y, Robledo D (2006) Carrageenan of *Eucheuma isiforme* (Solieriaceae, Rhodophyta) from Yucatán, Mexico. II. Seasonal variations in carrageenan and biochemical characteristics. Bot Mar 49(1):72–78. 10.1515/BOT.2006.009

[CR10] Gerardi, M. H., Lytle, B., & Zimmerman, M. C. (2015). The biology and troubleshooting of facultative lagoons: biology and troubleshooting of facultative lagoons. John Wiley & Sons, Incorporated. http://ebookcentral.proquest.com/lib/miami/detail.action?docID=1895856

[CR11] Gu M, Bai N, Zhang Y, Krogdahl Å (2016) Soybean meal induces enteritis in turbot *Scophthalmus maximus* at high supplementation levels. Aquaculture 464:286–295. 10.1016/j.aquaculture.2016.06.035

[CR12] Hao H, Fu M, Yan R, He B, Li M, Liu Q, Cai Y, Zhang X, Huang R (2019) Chemical composition and immunostimulatory properties of green alga Caulerpa racemosa var peltata. Food Agricult Immunol 30(1):937–54. 10.1080/09540105.2019.1646216

[CR13] Harwanto D, Saputro P, Susilowati T, Haditomo AHC, Windarto S (2020) Effect of different N:P ratios application on the cultivation media for the growth and fiber content of *Caulerpa racemosa* reared in tarpaulin ponds. AACL Bioflux 13(5):9

[CR14] Holdt SL, Kraan S (2011) Bioactive compounds in seaweed: functional food applications and legislation. J Appl Phycol 23(3):543–597. 10.1007/s10811-010-9632-5

[CR15] Jasmin MY, Syukri F, Kamarudin MS, Karim M (2020) Potential of bioremediation in treating aquaculture sludge: review article. Aquaculture 519:734905. 10.1016/j.aquaculture.2019.734905

[CR16] Kim JK, Stekoll M, Yarish C (2019a) Opportunities, challenges and future directions of open-water seaweed aquaculture in the United States. Phycologia (Oxford) 58(5):446–461. 10.1080/00318884.2019.1625611

[CR17] Kim JK, Kraemer G, Yarish C (2019b) Evaluation of the metal content of farm grown *Gracilaria tikvahiae* and *Saccharina latissima* from Long Island Sound and New York estuaries. Algal Res 40:1–9

[CR18] Laramore S, Baptiste R, Wills PS, Hanisak MD (2018) Utilization of IMTA-produced *Ulva lactuca* to supplement or partially replace pelleted diets in shrimp (*Litopenaeus vannamei*) reared in a clear water production system. J Appl Phycol 30(6):3603–3610. 10.1007/s10811-018-1485-3

[CR19] Leandro A, Pereira L, Gonçalves AMM (2019) Diverse applications of marine macroalgae. Mar Drugs 18(1):1. 10.3390/md18010017 31878264 10.3390/md18010017 PMC7024196

[CR20] Lohroff TJ, Gillette PR, Close HG, Benetti DD, Stieglitz JD (2021) Evaluating the potential bioextractive capacity of South Florida native macroalgae *Agardhiella subulata* for use in integrated multi-trophic aquaculture (IMTA). Aquaculture 544:737091. 10.1016/j.aquaculture.2021.737091

[CR21] Long ER, Sloane GM, Scott GI, Thompson B, Carr RS, Biedenback J, Wade TL, Presley BJ, Scott KJ, Mueller C, Brecken-Fols G, Albrecht B, Anderson JW, Chandler GT (1999). Magnitude and extent of chemical contamination and toxicity in sediments of Biscayne Bay and vicinity. NOAA/NOS CCMA 141. Technical Memorandum. National Oceanic and Atmospheric Administration, Silver Spring, MD, USA.

[CR22] Lozano I, Wacyk JM, Carrasco J, Cortez-san Martín MA (2016) Red macroalgae *Pyropia columbina* and *Gracilaria chilensis*: sustainable feed additive in the *Salmo salar* diet and the evaluation of potential antiviral activity against infectious salmon anemia virus. J Appl Phycol 28(2):1343–1351. 10.1007/s10811-015-0648-8

[CR23] Mac Monagail M, & Morrison L (2019). Arsenic speciation in a variety of seaweeds and associated food products. In Comprehensive Analytical Chemistry (Vol. 85, pp. 267–310). Elsevier.

[CR24] Magdugo RP, Terme N, Lang M, Pliego-Cortés H, Marty C, Hurtado AQ, Bedoux G, Bourgougnon N (2020) An analysis of the nutritional and health values of *Caulerpa racemosa* (Forsskål) and *Ulva fasciata* (Delile)—two Chlorophyta collected from the Philippines. Molecules 25(12):2901. 10.3390/molecules25122901 32599734 10.3390/molecules25122901 PMC7356146

[CR25] Millero FJ, Prieto FJ (2002) The values of pK1 + pK2 for the dissociation of carbonic acid in seawater. Geochim Cosmochim Acta 66(14):2529–2540. 10.1016/S0016-7037(02)00855-4

[CR26] Moreira A, Cruz S, Marques R, Cartaxana P (2022) The underexplored potential of green macroalgae in aquaculture. Rev Aquacult 14(1):5–26. 10.1111/raq.12580

[CR27] Narvarte B, Hinaloc L, Gonzaga S, Roleda M (2025) Exploring the suitable extractive species in an IMTA: inorganic nutrient removal from mariculture effluents by commercially important marine macroalgae. Bot Mar 68(4):403–415. 10.1515/bot-2025-0014

[CR28] National Institute of Health (2022a) Office of Dietary Supplements—magnesium. Retrieved June 3, 2023, from https://ods.od.nih.gov/factsheets/Magnesium-HealthProfessional/

[CR29] National Institute of Health (2022b) Office of Dietary Supplements—potassium. Retrieved June 4, 2023, from https://ods.od.nih.gov/factsheets/Potassium-HealthProfessional/

[CR30] National Institute of Health (2023a) Office of Dietary Supplements—omega-3 fatty acids. Retrieved June 3, 2023, from https://ods.od.nih.gov/factsheets/Omega3FattyAcids-HealthProfessional/

[CR31] National Institute of Health (2023b) Office of Dietary Supplements—iron. Retrieved June 4, 2023, from https://ods.od.nih.gov/factsheets/Iron-HealthProfessional/

[CR32] National Institute of Health (2024) Office of Dietary Supplements—calcium. Retrieved June 4, 2023, from https://ods.od.nih.gov/factsheets/Calcium-HealthProfessional/

[CR33] National Sea Grant Law Center (2023) FDA Classification of Seaweed. University of Mississippi. https://nsglc.olemiss.edu/projects/regulatingseaweed/files/fda-classification-of-seaweed.pdf

[CR34] Neori A, Chopin T, Troell M, Buschmann AH, Kraemer GP, Halling C, Shpigel M, Yarish C (2004) Integrated aquaculture: rationale, evolution and state of the art emphasizing seaweed biofiltration in modern mariculture. Aquaculture 231(1):361–391. 10.1016/j.aquaculture.2003.11.015

[CR35] Pádua M, Fontoura PSG, Mathias AL (2004) Chemical composition of *Ulvaria oxysperma* (kützing) bliding, *Ulva lactuca* (linnaeus) and *Ulva fascita* (delile). Braz Arch Biol Technol 47(1):49–55. 10.1590/S1516-89132004000100007

[CR36] Paul NA, de Nys R (2008) Promise and pitfalls of locally abundant seaweeds as biofilters for integrated aquaculture. Aquaculture 281(1):49–55. 10.1016/j.aquaculture.2008.05.024

[CR37] Peñalver R, Lorenzo JM, Ros G, Amarowicz R, Pateiro M, Nieto G (2020) Seaweeds as a functional ingredient for a healthy diet. Mar Drugs 18(6):6. 10.3390/md18060301 10.3390/md18060301 PMC7345263 32517092

[CR38] Perini V, Bracken M (2014) Nitrogen availability limits phosphorus uptake in an intertidal macroalga. Oecologia. 10.1007/s00442-014-2914-x 24615494 10.1007/s00442-014-2914-x

[CR39] Popp BN, Laws EA, Bidigare RR, Dore JE, Hanson KL, Wakeham SG (1998) Effect of phytoplankton cell geometry on carbon isotopic fractionation. Geochim Cosmochim Acta 62(1):69–77

[CR40] Rawiwan P, Peng Y, Paramayuda IGPB, Quek SY (2022) Red seaweed: a promising alternative protein source for global food sustainability. Trends Food Sci Technol 123:37–56. 10.1016/j.tifs.2022.03.003

[CR41] Ross FWR, Boyd PW, Filbee-Dexter K, Watanabe K, Ortega A, Krause-Jensen D, Lovelock C, Sondak CFA, Bach LT, Duarte CM, Serrano O, Beardall J, Tarbuck P, Macreadie PI (2023) Potential role of seaweeds in climate change mitigation. Sci Total Environ 885:163699. 10.1016/j.scitotenv.2023.163699 37149169 10.1016/j.scitotenv.2023.163699

[CR42] Rust MB, Amos KH, Bagwill AL, Dickhoff WW, Juarez LM, Price CS, Morris JA, Rubino MC (2014) Environmental performance of marine net-pen aquaculture in the United States. Fisheries 39(11):508–524. 10.1080/03632415.2014.966818

[CR43] Saito MA, Goepfert TJ, Ritt JT (2008) Some thoughts on the concept of colimitation: three definitions and the importance of bioavailability. Limnol Oceanogr 53(1):276–290. 10.4319/lo.2008.53.1.0276

[CR44] Shaughnessy BK, Jackson BP, Byrnes JEK (2023) Evidence of elevated heavy metals concentrations in wild and farmed sugar kelp (*Saccharina latissima*) in New England. Sci Rep 13:1–14. 10.1038/s41598-023-44685-4 37848595 10.1038/s41598-023-44685-4 PMC10582040

[CR45] Shpigel M, Guttman L, Shauli L, Odintsov V, Ben-Ezra D, Harpaz S (2017) *Ulva lactuca* from an integrated multi-trophic aquaculture (IMTA) biofilter system as a protein supplement in gilthead seabream (*Sparus aurata*) diet. Aquaculture 481:112–118. 10.1016/j.aquaculture.2017.08.006

[CR46] Shuuluka D, Bolton JJ, Anderson RJ (2013) Protein content, amino acid composition and nitrogen-to-protein conversion factors of *Ulva rigida* and *Ulva capensis* from natural populations and *Ulva lactuca* from an aquaculture system, in South Africa. J Appl Phycol 25(2):677–685. 10.1007/s10811-012-9902-5

[CR47] Sigman DM, Casciotti KL (2001) Nitrogen isotopes in the ocean. Encycloped Ocean Sci 3:1884–1894

[CR48] Tanaka Y, Ashaari A, Mohamad FS, Lamit N (2020) Bioremediation potential of tropical seaweeds in aquaculture: low-salinity tolerance, phosphorus content, and production of UV-absorbing compounds. Aquaculture 518:734853. 10.1016/j.aquaculture.2019.734853

[CR49] Trushenski JT, Kasper CS, Kohler CC (2006) Challenges and opportunities in finfish nutrition. N Am J Aquacult 68(2):122–140

[CR50] U.S. Food and Drug Administration (2024a) Mercury in food. https://www.fda.gov/food/environmental-contaminants-food/mercury-food

[CR51] U.S. Food and Drug Administration (2024b) Cadmium in food and foodwares. https://www.fda.gov/food/environmental-contaminants-food/cadmium-food-and-foodwares

[CR52] U.S. Food and Drug Administration (2024c) Lead in food and foodwares. https://www.fda.gov/food/environmental-contaminants-food/lead-food-and-foodwares

[CR53] Yaich H, Garna H, Besbes S, Paquot M, Blecker C, Attia H (2011) Chemical composition and functional properties of *Ulva lactuca* seaweed collected in Tunisia. Food Chem 128(4):895–901. 10.1016/j.foodchem.2011.03.114

[CR54] Zhou Y, Yang H, Hu H, Liu Y, Mao Y, Zhou H, Xu X, Zhang F (2006) Bioremediation potential of the macroalga *Gracilaria lemaneiformis* (Rhodophyta) integrated into fed fish culture in coastal waters of north China. Aquaculture 252(2–4):264–276. 10.1016/j.aquaculture.2005.06.046

[CR55] Zhu Y, Wang N, Wu Z, Chen S, Luo J, Christakos G, Wu J (2025) The role of seaweed cultivation in integrated multi-trophic aquaculture (IMTA): the current status and challenges. Rev Aquacult 17:1–15. 10.1111/raq.70042

