# Supplementary material for: Evaluation of native macroalgae species of the Southeast U.S. and Caribbean for use in integrated multi-trophic aquaculture (IMTA)
Source: Aquac Int. 2026 Feb 10;34(2):70. doi: 10.1007/s10499-026-02441-1 (PMC12939857; doi:10.1007/s10499-026-02441-1)
Supplement: Supplementary file 1 — Supplementary file1 (DOCX 16 kb) [file 10499_2026_2441_MOESM1_ESM.docx]

Supplementary Data:

Table 2 cont: The sample size of Agardhiella subulata (As) was 90 g dry weight (DW), Caulerpa racemosa (Cr) was 21 g DW, Gracilaria caudata (Gc) was 46 g DW, and Ulva lactuca (Ul) was 137 g DW.

Equations of lines depicted in Figure 3: *Agardhiella subulata* polynomial (2nd order) relationships in growth of seaweed over time and TAN removal (%) over time: y = 0.0226x^2^ + 0.4612x + 0.2527, R² = 0.9895 and y = -0.0724x^2^ + 2.7182x + 76.364, R² = 0.6948 respectively. *Caulerpa racemosa* polynomial (2nd order) relationships in growth of seaweed over time and TAN removal (%) over time: y = 0.0011x^2^ - 0.0988x + 2.8138, R² = 0.5602 and y = 0.6973x^2^ - 11.649x + 37.083, R² = 0.9874 respectively. *Gracilaria caudata* polynomial (2nd order) relationships in growth of seaweed over time and TAN removal (%) over time: y = -0.0057x^2^ + 0.2062x + 0.3938, R² = 0.9358 and y = -0.2369x^2^ + 6.0133x + 30.901, R² = 0.394 respectively. *Ulva lactuca* polynomial (2nd order) relationships in growth of seaweed over time and TAN removal (%) over time: y = 0.0651x^2^ - 0.4356x + 0.5765, R² = 0.994 and y = -0.3908x^2^ + 14.867x - 24.783, R² = 0.9045 respectively.

Equations of lines depicted in Figure 4: *Agardhiella subulata* polynomial (2nd order) relationships in phosphate of seaweed over time and fish effluent phosphate concentrations over time: y = 0.0072x^2^ - 0.2003x + 1.6844, R² = 0.7443 and y = 0.0091x^2^ - 0.1993x + 1.5088, R² = 0.5787 respectively. *Caulerpa racemosa* polynomial (2nd order) relationships in phosphate of seaweed over time and fish effluent phosphate concentrations over time: y = -0.0023x^2^ + 0.0146x + 0.2838, R² = 0.4473 and y = 0.0005x^2^ - 0.0271x + 0.3637, R² = 0.158 respectively. *Gacilaria cuadata* polynomial (2nd order) relationships in phosphate of seaweed over time and fish effluent phosphate concentrations over time: y = 0.0048x^2^ - 0.1532x + 1.5228, R² = 0.6533 and y = 0.0091x^2^ - 0.1993x + 1.5088, R² = 0.5787 respectively. *Ulva lactuca* polynomial (2nd order) relationships in phosphate of seaweed over time and fish effluent phosphate concentrations over time: y = 0.0054x^2^ - 0.1834x + 1.7141, R² = 0.8144 and y = 0.0091x^2^ - 0.1993x + 1.5088, R² = 0.5787 respectively.

Equations of lines depicted in Figure 5: *Agardhiella subulata* polynomial (2nd order) relationships in fish pH over time and pH of seaweed over time: y = 0.0013x^2^ - 0.0243x + 8.0658, R² = 0.3709 and y = -0.0019x^2^ + 0.0462x + 8.0863, R² = 0.5197 respectively. *Caulerpa racemosa* polynomial (2nd order) relationships in fish pH over time and pH of seaweed over time: y = -0.0009x^2^ + 0.0138x + 7.9438, R² = 0.1167 and y = 0.0005x^2^ - 0.0052x + 8.0979, R² = 0.2533 respectively. *Gracilaria caudata* polynomial (2nd order) relationships in fish pH over time and pH of seaweed over time: y = 0.0013x^2^ - 0.0243x + 8.0658, R² = 0.3709 and y = -0.0002x^2^ + 0.0118x + 8.0834, R² = 0.484 respectively. *Ulva lactuca* polynomial (2nd order) relationships in fish pH over time and pH of seaweed over time: y = 0.0013x^2^ - 0.0243x + 8.0658, R² = 0.3709 and y = 0.0006x^2^ + 0.0358x + 7.9736, R² = 0.7307 respectively.

Equations depicted in Figure 6: *Agardhiella subulata* polynomial (2nd order) relationships in CO₂ concentration of seaweed effluent over time and CO₂ concentration of fish effluent over time: y = 0.0028x^2^ - 0.0591x + 0.6096, R² = 0.56 and y = -0.0023x^2^ + 0.0452x + 0.712, R² = 0.3302 respectively. *Caulerpa racemosa* polynomial (2nd order) relationships in CO₂ concentration of seaweed effluent over time and CO₂ concentration of fish effluent over time: y = -0.0005x^2^ - 0.0016x + 0.6254, R² = 0.5564 and y = 0.0031x^2^ - 0.05x + 0.9863, R² = 0.1838 respectively. *Gracilaria caudata* polynomial (2nd order) relationships in CO₂ concentration of seaweed effluent over time and CO₂ concentration of fish effluent over time: y = 0.0006x^2^ - 0.0203x + 0.6349, R² = 0.3424 and y = -0.0023x^2^ + 0.0452x + 0.712, R² = 0.3302 respectively. *Ulva lactuca* polynomial (2nd order) relationships in CO₂ concentration of seaweed effluent over time and CO₂ concentration of fish effluent over time: y = 0.0005x^2^ - 0.0535x + 0.7949, R² = 0.8045 and y = -0.0023x^2^ + 0.0452x + 0.712, R² = 0.3302 respectively.

Equations depicted in Figure 7: *Agardhiella subulata* polynomial (2nd order) relationships in growth of seaweed over time: y = 0.0262x^2^ + 0.3791x + 0.3816, R² = 0.9798. *Caulerpa racemosa* polynomial (2nd order) relationships in growth of seaweed over time: y = 0.0037x^2^ - 0.1337x + 2.8285, R² = 0.5027. *Gracilaria caudata* polynomial (2nd order) relationships in growth of seaweed over time: y = -0.0092x^2^ + 0.2163x + 0.3585, R² = 0.7381. *Ulva lactuca* polynomial (2nd order) relationships in growth of seaweed over time: y = 0.0533x^2^ - 0.3449x + 0.486, R² = 0.9773.

Equations of lines depicted in Figure 8: *Agardhiella subulata* polynomial (2nd order) relationships in Carbon Nitrogen ratio: y = -0.0347x^2^ + 0.5312x + 8.3558, R² = 0.607. *Caulerpa racemosa* polynomial (2nd order) relationships in Carbon Nitrogen ratio: y = 0.0146x^2^ - 0.2842x + 10.621, R² = 0.9769. *Gracilaria caudata* polynomial (2nd order) relationships in Carbon Nitrogen ratio: y = 0.0199x^2^ - 0.1383x + 14.241, R² = 0.9977. *Ulva lactuca* polynomial (2nd order) relationships in Carbon Nitrogen ratio: y = 0.0654x^2^ - 0.5783x + 13.806, R² = 0.6364.
